# Vancomycin susceptibility in methicillin-resistant *Staphylococcus aureus* is mediated by YycHI activation of the WalRK essential two-component regulatory system

**DOI:** 10.1038/srep30823

**Published:** 2016-09-07

**Authors:** David R. Cameron, Jhih-Hang Jiang, Xenia Kostoulias, Daniel J. Foxwell, Anton Y. Peleg

**Affiliations:** 1 Department of Microbiology, Monash University, Melbourne, Australia; 2 Department of Infectious Diseases, Alfred Hospital and Monash University, Melbourne, Australia

## Abstract

The treatment of infections caused by methicillin-resistant *Staphylococcus aureus* is complicated by the emergence of strains with intermediate-level resistance to vancomycin (termed VISA). We have characterised a molecular pathway involved in the *in vivo* evolution of VISA mediated by the regulatory proteins YycH and YycI. In contrast to their function in other bacterial species, we report a positive role for these auxiliary proteins in regulation of the two-component regulator WalRK. Transcriptional profiling of *yycH* and *yycI* deletion mutants revealed downregulation of the ‘WalRK regulon’ including cell wall hydrolase genes *atlA* and *sle1*, with functional autolysis assays supporting these data by showing an impaired autolytic phenotype for each deletion strain. Using bacterial-two hybrid assays, we showed that YycH and YycI interact, and that YycHI also interacts with the sensor kinase WalK, forming a ternary protein complex. Mutation to YycH or YycI associated with clinical VISA strains had a deleterious impact on the YycHI/WalK complex, suggesting that the interaction is important for the regulation of WalRK. Taken together, we have described a novel antibiotic resistance strategy for the human pathogen *S. aureus*, whereby YycHI mutations are selected for *in vivo* leading to reduced WalRK activation, impaired cell wall turnover and ultimately reduced vancomycin efficacy.

Methicillin-resistant *Staphylococcus aureus* (MRSA) is a frequent cause of both hospital- and community-acquired infections, and invasive disease such as bacteremia is associated with high mortality^1^. The increased reliance on the glycopeptide antibiotic vancomycin has resulted in the development of strains with reduced vancomycin susceptibility, termed vancomycin-intermediate *S. aureus* (VISA)^2^. VISA strains typically emerge as a result of prolonged vancomycin treatment during complex infections such as endocarditis and osteomyelitis, and importantly, infections with VISA strains are associated with persistent bacteremia and poor clinical outcomes^3^,^4^.

Describing the genetic correlates contributing to VISA has remained challenging. Unlike MRSA, which emerged as a result of the horizontal acquisition of *mecA* encoding an alternative penicillin binding protein (PBP2a), no molecular ‘silver bullet’ is responsible for the development of VISA^5^. A landmark study by Mwangi *et al*. applied comparative genomics to a series of vancomycin-exposed clinical strains and showed that incremental increases in the minimum inhibitory concentration (MIC) of vancomycin correlated with cumulative mutations in diverse genetic pathways^6^. Subsequent studies have described an abundance of point mutations in regulatory loci that moderate cell wall metabolism including two-component regulatory systems (TCRS, eg. *walRK*, *graSR* and *vraSR*) and differential expression of the ‘cell wall stimulon’ in VISA isolates^7^,^8^,^9^,^10^,^11^. In addition to cell wall thickening, VISA is associated with an altered surface protein profile, enhanced capsule and *agr* dysfunction, each of which likely affects host-pathogen interactions^12^. It has been postulated that these changes contribute to immune evasion and the bacterial persistence that is observed clinically^12^,^13^.

The WalRK TCRS is a critical regulator of cell wall metabolism in *S. aureus*^14^. Upon activation by the WalK sensor histidine kinase, the WalR response regulator binds to the promoter sequence and increases the expression of multiple staphylococcal autolysins including *atlA, sle1* and *lytM*^14^,^15^. Point mutations in the *walRK* operon are frequently described for VISA and have also been identified in strains resistant to a second last-line antibiotic, daptomycin^16^,^17^. In *Bacillus subtilis*, YycH and YycI negatively regulate the WalRK ortholog, YycFG^18^,^19^. It appears that YycHI form a complex with the sensor histidine kinase YycG, preventing it from activating its cognate response regulator, YycF^18^,^19^. Thus far, the function of YycHI in *S. aureus* and its contribution to the *in vivo* evolution of VISA is yet to be characterised.

In the present study, we have described a role for YycHI to vancomycin susceptibility in *S. aureus*. Chromosomal deletion of *yycH* and/or *yycI* from a clinical vancomycin-sensitive *S. aureus* (VSSA) strain was sufficient to produce a heterogenous vancomycin-intermediate (hVISA) phenotype. Transcriptional profiling of the *yycH* and *yycI* mutants revealed downregulation of the WalRK ‘regulon’, which was corroborated by reduced autolysis. These data are in contrast to that seen in *Bacillus* sp. and suggest a role for YycHI in activation of the WalRK TCRS. Using a bacterial two-hybrid approach, we have proposed that complex formation between YycHI and WalK is important for WalRK regulation, and that *yycHI* mutation in clinical *S. aureus* strains contributes to VISA by disrupting this interaction thereby reducing WalRK activity and impairing cell wall turnover.

## Results

### Genetic analysis of *yycHI* mutations in clinical VISA strains

**Figure 1:**
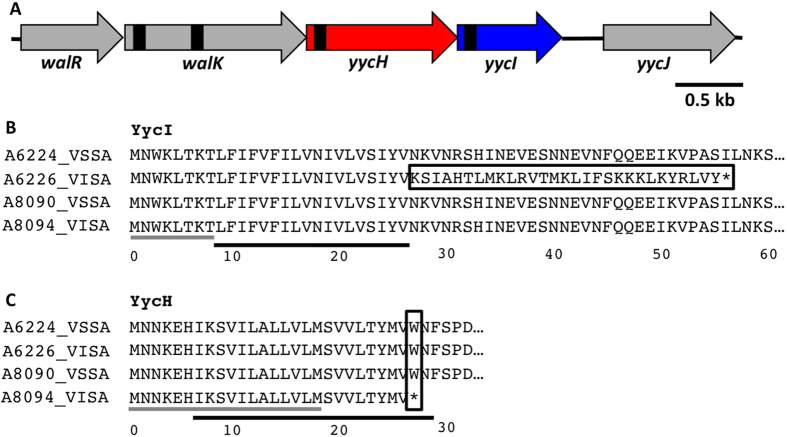
Genetic organisation of the *walRK* operon in *S. aureus*. (**A**) The *walRK* two-component regulatory system is encoded directly upstream of *yycH*, *yycI* and *yycJ.* Predicted transmembrane domains are presented in black. (**B**) A 262 amino acid protein is coded for by the consensus sequence of *yycI* in *S. aureus*. Mutation to *yycI* in clinical VISA isolate A6226 generates a frameshift (black box) and truncation of the protein to 56 amino acids. (**C**) A 453 amino acid protein is coded for by the consensus sequence of *yycH* in *S. aureus.* A single nucleotide polymorphism in clinical VISA strain A8094 generates a premature stop codon at the 27^th^ amino acid (black box). Black lines represent predicted transmembrane domains for the consensus sequence of each protein. Grey lines represent native residues that remain in each in-frame deletion strain.

We previously performed whole-genome sequencing of multiple, clinically-derived VISA isolates to characterise the genetic mechanisms of the *in vivo* evolution of reduced susceptibility to vancomycin in *S. aureus*^9^. We identified a novel deletion mutation within *yycI*, which belongs to the *walRK/yycHIJ* operon. In *S. aureus*, this operon consists of *walR*, which codes for a response regulator, *walK*, which codes for a sensor histidine kinase, *yycH* and *yycI*, which encode putative regulatory proteins and *yycJ*, which codes for a 5′-3′ exonuclease involved in mismatch repair^20^ (Fig. 1A). The *yycI* mutation was one of 13 found between the VSSA progenitor strain A6224 and VISA strain A6226 that emerged after prolonged vancomycin treatment^9^. The four-nucleotide deletion (ATAA from the position 79–82) resulted in a predicted frameshift from the 27^th^ amino acid and truncation of the protein from 262 amino acids in the VSSA strain (A6224) to 56 amino acids in the VISA strain (A6226) (Fig. 1B). No mutation was observed for the upstream protein, YycH (Fig. 1C). Intriguingly, in a separate study, Mwangi *et al*. identified 35 mutations between VISA strain JH9 (A8094 in this study) and the VSSA progenitor strain JH1 (A8090 in this study) including a single nucleotide polymorphism (SNP) in *yycH*^6^. The G to A substitution at the 81^st^ nucleotide base was predicted to change the tryptophan at position 27 to a premature stop codon, truncating the protein to ~5% of its original length (453 amino acids) (Fig. 1C). No YycI mutation was observed in the VISA strain (A8094) (Fig. 1B). DNA sequencing confirmed the *yycHI* mutations in A6226 and A8094. Given the degree of truncation, and the predicted role for the proteins in WalRK regulation, we hypothesized that *yycHI* mutation likely contributes to reduced vancomycin susceptibility in clinical VISA strains.

### YycH and YycI influence vancomycin susceptibility

**Figure 2:**
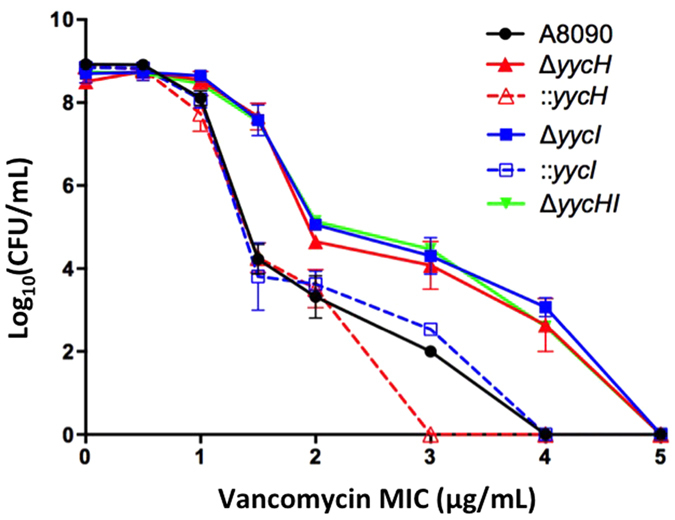
Vancomycin susceptibility profiles of A8090 and *yycHI* mutant strains. Vancomycin heteroresistance was assessed for A8090, deletion mutants (Δ*yycH,* Δ*yycI* and Δ*yycHI*) and gene reconstituted strains (*::yycH* and*::yycI*) using population analysis profiling on heart infusion agar supplemented with increasing concentrations of vancomycin. Data are expressed as mean ± SEM.

To determine the exact contribution of *yycHI* to the development of VISA independent of other mutations, chromosomal deletions of *yycH* (A8090Δ*yycH*), *yycI* (A8090Δ*yycI*) and *yycHI* (A8090Δ*yycHI*) were generated within the genetic context of VSSA clinical isolate, A8090. Vancomycin susceptibility was assessed using Etest and population analysis profiling (PAP). A8090Δ*yycH* and A8090Δ*yycI* each showed a reduction in vancomycin susceptibility, with a MIC of vancomycin increasing from 1.0 μg/mL to a maximum of 3.0 μg/mL (range 2.0–3.0 µg/ml) for both mutants, which is in the non-susceptible range (>2 μg/mL) (Table 1), and has been associated with poor patient outcomes^21^. Notably, the MIC for vancomycin of the double mutant (A8090Δ*yycHI*) was no greater (Table 1) than for each of the single mutants, suggesting that both YycH and YycI proteins are equally essential for vancomycin susceptibility. PAP analyses are the gold standard for assessing VISA and heterogeneous VISA (hVISA), and provide a more sensitive quantification of resistant sub-populations. Each deletion strain had a greater number of cells tolerant to >2 μg/mL of vancomycin (Fig. 2), which defines the VISA phenotype and predicts for persistent bacteremia and complicated infection outcomes^3^,^22^.

Genetic reconstitution of *yycH* and *yycI in situ* returned the MIC for vancomycin to within the susceptible range (1.0 μg/mL, Table 1) and each reconstituted strain produced a similar vancomycin population profile to that of the vancomycin-susceptible parent strain (Fig. 2). Together, these data confirm that both YycH and YycI are important for vancomycin susceptibility in *S. aureus*.

Deletion of *yycH* or *yycI* from VSSA strain A8090 did not recreate the MIC of vancomycin for the clinical VISA isolate A8094 (8.0 μg/mL). This suggests that additional mutations in A8094 contribute to the VISA phenotype for this strain and supports the theory that VISA emerge as a result of cumulative mutations during prolonged vancomycin exposure. In addition, the *in vivo* evolution of VISA in A8094 and A6226 was associated with co-resistance to daptomycin in the absence of daptomycin exposure (Table 1). To determine if *yycHI* mutation contributed to the daptomycin-nonsusceptible phenotype of these isolates, daptomycin susceptibility testing was performed including PAP analyses. No change in susceptibility was observed for A8090Δ*yycH* or A8090Δ*yycI* when compared to A8090 (Table 1, PAP data not shown). It is likely that mutations present in A8094 and A6226 other than those in *yycHI* contributed to daptomycin resistance in these isolates.

### YycHI positively regulates WalRK in *S. aureus*

**Figure 3:**
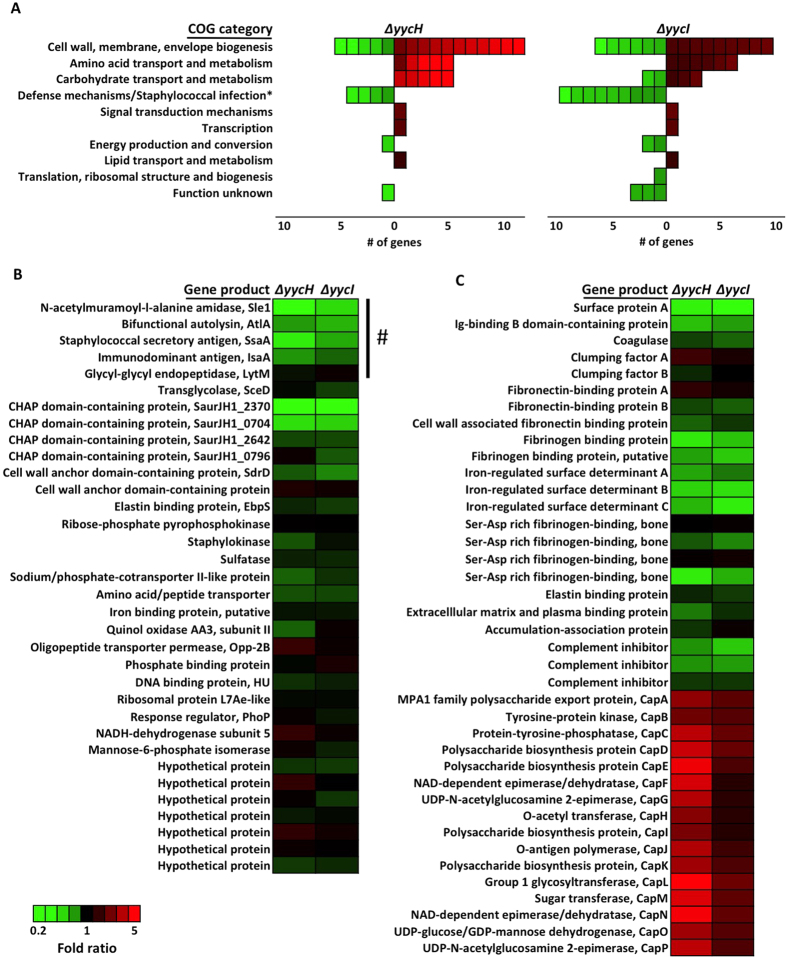
Transcriptional comparison of *yycH* and *yycI* deletion mutants. (**A**) RNA sequencing was used to generate lists of genes with differential expression in Δ*yycH* and Δ*yycI* compared to parental strain A8090. Each gene was assigned a cluster of orthologous groups (COG) category or Kyoto encyclopedia of genes and genomes (KEGG) pathway category (represented by *). Genes upregulated in each deletion strain are represented in red, whilst genes downregulated in each deletion strain are represented in green. Transcriptional profiles of Δ*yycH* and Δ*yycI* were highly similar. (**B**) Genes predicted to be regulated by WalRK are largely downregulated in Δ*yycH* and Δ*yycI.* Genes confirmed to be under the transcriptional control of WalRK are denoted by #^14^,^24^,^46^. Remaining genes are predicted to be under the control of WalRK due to the presence of an upstream putative WalR binding site^14^,^46^. (**C**) Genes involved in host-pathogen interaction were differential expressed in Δ*yycH* and Δ*yycI.* Genes encoding surface proteins, adhesins and complement inhibitors were mostly downregulated, whilst the capsule operon was strongly overexpressed in Δ*yycH* and Δ*yycI.*

To determine how YycHI contributes to antibiotic susceptibility and to understand the impact of these proteins upon WalRK, we compared the transcriptomes of A8090, Δ*yycH* and Δ*yycI* using RNA-Seq. Transcriptomes were sequenced using the Illumina sequencing platform yielding an average total read count of 6.67 million reads per sample. The transcriptomes of Δ*yycH* and Δ*yycI* were highly similar (only six differentially expressed genes representing 0.2% of the transcriptome [Supplementary Table S1]), suggesting that both proteins function within the same system and are equally important in *S. aureus* physiology (Fig. 3). When the transcriptome of Δ*yycH* or Δ*yycI* was compared to the progenitor strain A8090, a total of 42 genes showed altered expression (≥2-fold) in at least one of the deletion strains. These included 17 downregulated genes (Supplementary Table S2) and 25 upregulated genes (Supplementary Table S3). When each differentially expressed gene was grouped based on Clusters of Orthologous Groups (COG) or Kyoto encyclopedia of genes and genomes (KEGG) pathways, 40% of the genes were predicted to be involved in cell wall, membrane and envelope biosynthesis (Fig. 3A). Furthermore, 21% of genes were predicted to be involved in staphylococcal infection and defence (Fig. 3A). Quantitative digital PCR of select genes confirmed these RNA-Seq data (Supplementary Fig. S1).

WalR is a critical transcriptional regulator in *S. aureus* and has been shown to influence the expression of genes involved in cell wall turnover, which explains its role in the development of VISA, as well as a diverse range of genes important for amino acid biosynthesis, central metabolism and virulence^14^,^15^,^16^. However, experimental confirmation of WalR binding to the promoter sequence of specific genes and induction of their expression has only been confirmed for five genes, each of which encode for proteins with autolytic activity (*sle1*, *atlA*, *ssaA*, *isaA* and *lytM*)^14^,^15^. Based on work in *Bacillus*, we hypothesised that YycHI binds to WalK and alters its regulatory activity^18^,^19^. To determine the direction of YycHI-mediated regulation of WalRK in *S. aureus*, we interrogated our RNA-seq data for the expression of validated WalR regulated genes. We showed that except for *lytM*, the remaining four genes (*sle1*, *atlA*, *ssaA* and *isaA*) were downregulated in both the Δ*yycH* and Δ*yycI* deletion mutants when compared to their progenitor strain (Fig. 3B and Supplementary Table S2). Furthermore, whilst upstream WalR binding is yet to be confirmed, *walRK* induction increases the expression of four CHAP-domain containing amidase/peptidoglycan hydrolase genes (SaurJH1_0704, SaurJH1_0796, SaurJH1_2370 and SaurJH1_2642)^14^. Of these, SaurJH1_0704 and SaurJH1_2370 were significantly downregulated in Δ*yycH* and Δ*yycI* (Fig. 3B and Supplementary Table S2). We also showed, similar to that observed in *Bacillus*^19^, that YycH has a growth-phase dependent impact on gene expression that is most pronounced at early stationary compared to mid-exponential phase (Supplementary Fig. S2A). These transcriptomic changes suggest that in contrast to that seen in *Bacillus* sp., YycHI positively regulates the WalRK TCRS in *S. aureus.* Importantly, the transcription of *walRK* was not different for either Δ*yycH* or Δ*yycI* when compared to the progenitor strain A8090.

**Figure 4:**
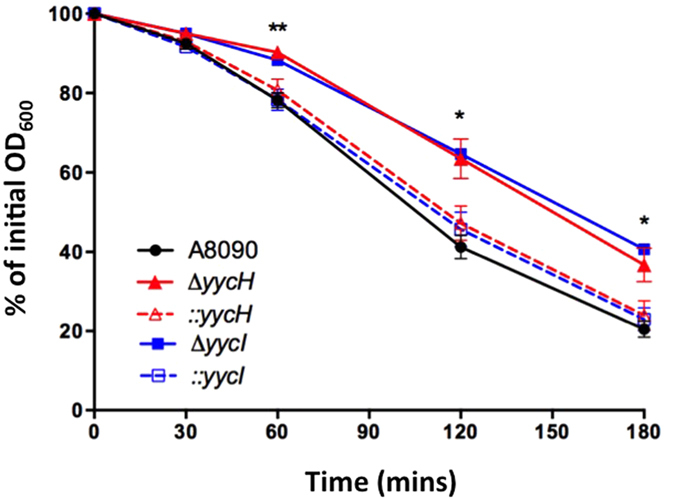
Deletion of *yycH* or *yycI* reduces autolysis. Autolysis was determined as the decline in OD_600_ over time at 30 °C in the presence of Triton X-100. Data are expressed as mean ± SEM (**P < 0.01, *P < 0.05).

To provide further support to the direction of WalRK regulation by YycHI in *S. aureus*, we performed functional autolysis assays. It is well established that WalRK is a regulator of autolysis in *S. aureus*^14^ and this is supported by the five validated WalR-regulated genes that all encode proteins with autolytic activity^14^,^15^. Using Triton X-100 induction, we compared the autolytic rate of Δ*yycH* and Δ*yycI* to their progenitor, A8090. Each deletion strain showed impaired autolysis as determined by a reduced decline in OD_600_ over time (Fig. 4). Gene reconstitution restored the autolytic rate back to that of the vancomycin-susceptible parent strain (A8090) (Fig. 4). Together, the observed differential gene expression and the functional impact on autolysis suggests that YycHI acts as a positive regulator of WalRK in *S. aureus*. Deletion of either YycH or YycI leads to downregulation of WalR-regulated genes and WalR-mediated autolysis, which likely contributes to reduced susceptibility to the cell-wall acting antibiotic, vancomycin.

### Transcriptional effects of *yycHI* deletion upon genes associated with host-pathogen interaction

In addition to regulating cell wall turnover, recent reports have shown that WalRK indirectly controls the expression of a number of virulence factors via activation of the SaeSR TCRS^15^. Fittingly, our transcriptomic data highlighted down-regulation of multiple genes that are involved in host-pathogen interaction, a number of which are indirectly regulated by WalR (Fig. 3C)^15^. These included immunoglobulin-binding proteins encoded by *spa* and *sbi,* which were downregulated in both Δ*yycH* and Δ*yycI*. In addition, fibrinogen-binding proteins encoded by *efb* and SaurJH1_1235 were less abundant in Δ*yycH* and Δ*yycI*, and excreted factors *scn* and SaurJH1_1240 encoding staphylococcal complement inhibitors were significantly downregulated in Δ*yycI* (Supplementary Table S2). In contrast, the entire capsule operon was overexpressed in both Δ*yycH* and Δ*yycI* (Fig. 3C), which is commonly reported for VISA strains^12^.

### YycH, YycI and WalK form a ternary protein complex

**Figure 5:**
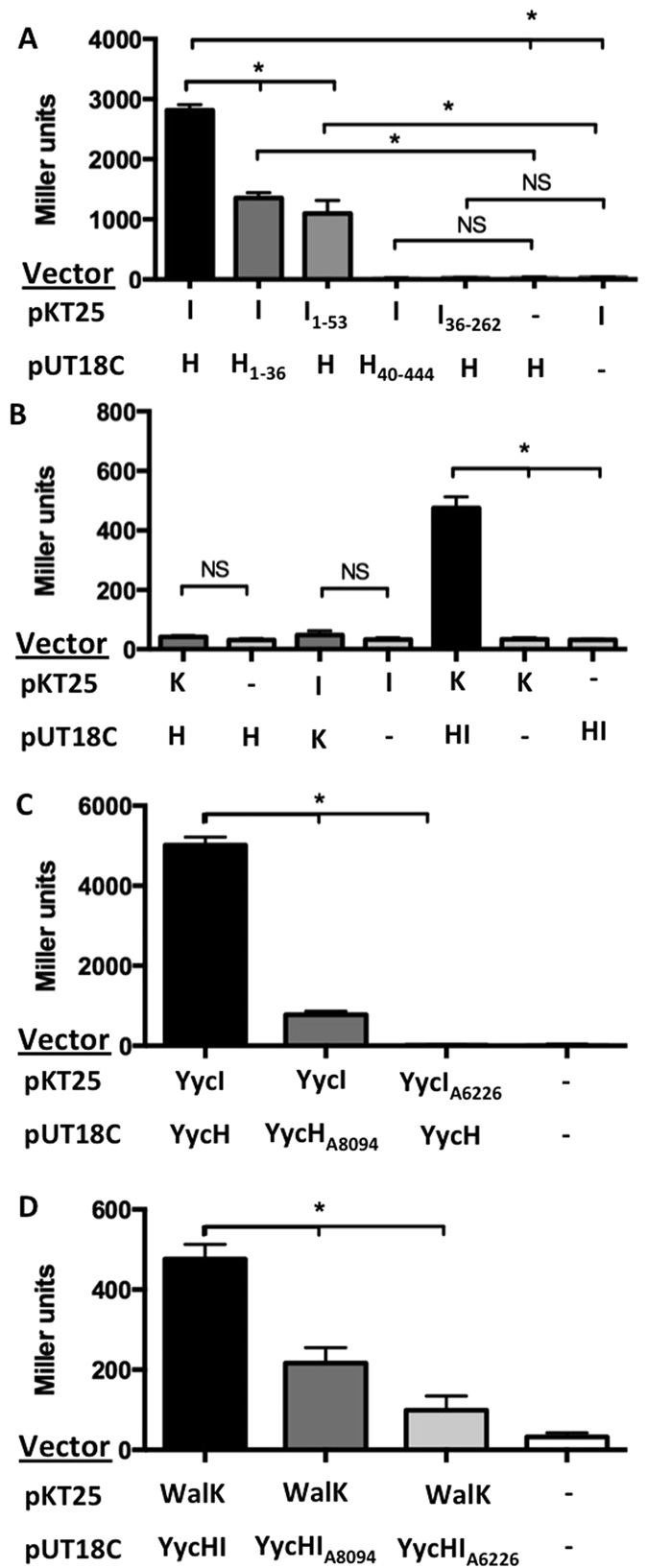
YycH, YycI and WalK interactions as determined by bacterial-two-hybrid analysis. (**A**) β-galactosidase assays were performed for *E. coli* co-expressing bacterial two-hybrid fusions of full-length YycH (H) and YycI (I) as well as fragments containing the transmembrane domain of each protein (H_1–36_ and I_1–53_) or lacking each respective transmembrane domain (H_40–444_ and I_36–262_). (**B**) β-galactosidase assays were also performed for *E. coli* expressing combinations of WalK (K), YycH (H), YycI (I) and YycHI (HI). (**C**) YycH mutation associated with VISA strain A8094 (YycH_A8094_) reduced the strength of interaction with YycI, and YycI mutation associated with VISA strain A6226 (YycI_A6226_) reduced the strength of interaction with YycH as determined by β-galactosidase activity. (**D**) Similarly, mutations associated with A8094 (YycHI_A8094_) and A6226 (YycHI_A6226_) reduced the strength of the complex formed between YycH, YycI and WalK. No interaction controls were generated using pKT25 or pUT18C without gene inserts (represented by -). Data are presented as mean ± SEM (*P < 0.05).

Thus far little is known about the staphylococcal YycH and YycI proteins and their interactions. Work in *B. subtilis* has showed that YycH and YycI are tethered to the cytoplasmic membrane and physically interact at their respective transmembrane domains (TMDs)^18^. Using a bacterial-two hybrid system, we assessed the interactions between YycH and YycI from *S. aureus*. Full-length YycH was found to interact with full-length YycI using this approach (Fig. 5A). We observed that except for the TMDs of each protein (YycI_1–53_ and YycH_1–36_), the remainder of the protein was dispensable for their interaction (Fig. 5A). YycH/YycI constructs lacking TMDs did not interact with their respective full-length binding partners (Fig. 5A). These data suggest that staphylococcal YycHI interactions are mediated by the TMDs.

To determine whether YycH/YycI interact with the sensor kinase WalK, we first tested them individually. Similar to our observations for vancomycin susceptibility, YycH or YycI alone were not sufficient to produce a quantifiable interaction with WalK (Fig. 5B), suggesting that YycH and YycI are required as a protein complex before they can adequately interact with WalK. To test this hypothesis, full-length YycH, YycI and WalK were co-expressed in *Escherichia coli* BTH101; a quantifiable interaction was observed (Fig. 5B).

### *yycHI* mutations associated with clinical VISA strains disrupt the interaction between YycH, YycI and WalK

**Figure 6:**
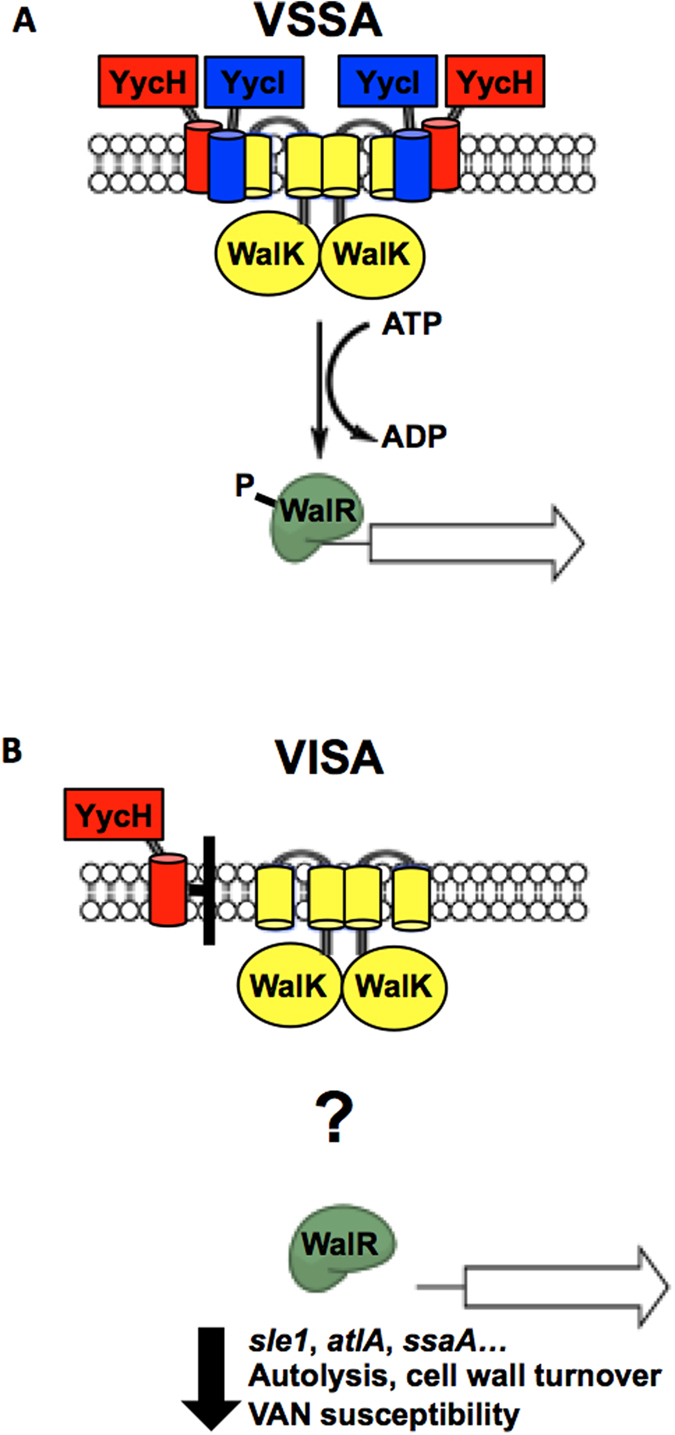
Proposed model for the impact of YycHI mutation upon the activation of WalKR in the context of VISA. (**A**) In vancomycin-sensitive strains, YycH and YycI physically interact with the sensor kinase to activate the WalRK TCRS. WalR is phosphorylated by WalK leading to transcription of target genes. (**B**) Mutation to either YycH or YycI occurring in clinical VISA strains has a negative impact on complex formation, resulting in reduced expression of WalR-regulated genes via an unknown mechanism. Reduced WalR activity contributes to reduced autolysis, impaired cell wall turnover and ultimately reduced vancomycin (VAN) susceptibility.

In addition to *yycHI* mutations described in this report, numerous diverse *walK* point mutations have been described in VISA strains^16^,^17^. To date, it is unclear how these mutations impact on the activity of WalRK or the relationship with YycHI. As such, we next investigated the impact of VISA associated *walK/yycHI* mutations upon the interaction between YycH, YycI and WalK using our bacterial two-hybrid system. WalK proteins containing amino acid substitutions associated with diverse VISA isolates were assessed including G233D from JKD6008, Q371Δ from A8392, and R263C/S273N from A8118 (Table 1). These WalK mutations had no effect on the interaction with YycHI (data not shown). We next assessed the impact of *yycHI* mutations from the clinical VISA strains A8094 and A6226 (Fig. 1). The interaction between YycH and YycI was reduced 6-fold when the mutated YycH from A8094 was assessed, and the interaction was abolished when the mutated YycI from A6226 was included in the analysis (Fig. 5C). Most importantly, the interaction with the kinase, WalK was significantly reduced when mutated YycH or YycI proteins from either A8094 or A6226 were included (Fig. 5D). Together, these data suggest that reduced vancomycin susceptibility in our clinical VISA strains is due to impaired interaction of the YycHI protein complex with WalK, leading to reduced expression of the WalR regulon and reduced WalR-mediated autolysis. A proposed model for this is shown in Fig. 6.

## Discussion

Defining the complete molecular pathway to reduced vancomycin susceptibility in *S. aureus* has remained challenging owing to the heterogeneous nature of the genetic mutations associated with VISA. We, and others have used whole-genome sequencing of clinical isolates to guide targeted genetic manipulation experiments to determine the exact contribution of select genes to reduced susceptibility to vancomycin, independent of other mutations^9^,^16^. By engineering *yycH* and *yycI* deletions from a vancomycin-susceptible strain, we have described for the first time the contribution of these genes to the development of reduced vancomycin susceptibility and we confirmed their importance by restoring the vancomycin-susceptible phenotype via gene reconstitution (Fig. 2).

The function of YycH and YycI in *S. aureus* is typically inferred from their role in *Bacillus* sp. where they negatively regulate the WalRK ortholog YycFG^18^,^19^. WalRK/YycFG is highly conserved across low GC content gram-positive bacteria, and is essential in *S. aureus* most likely due to its crucial role in regulating cell wall metabolism^23^,^24^. Due to its role in cell wall maintenance, it is not surprising that WalRK mutation is commonly described for VISA^16^,^17^. Two distinct mechanisms have been identified that infer a role for WalRK in the evolution of VISA. Firstly, IS256 insertion in the promoter of the operon has been shown to reduce vancomycin susceptibility and secondly, point mutations within *walK* and *walR* contribute to vancomycin tolerance in clinical VISA strains^16^,^25^,^26^. What remains unclear, however, is the impact of these mutations upon the activity of WalRK. Jansen *et al*. showed that IS256 insertion led to increased expression of *walRK* whereas more recently, McEvoy *et al*. showed that upstream IS256 insertion decreased expression of the TCRS^25^,^26^. Similarly, transcriptional profiling of VISA-associated *walK* and *walR* point mutants revealed no clear directionality for the activity of the TCRS based on contrasting expression of *isaA* and *atlA*, each of which are induced by WalR^16^. In this study, we have described a third mechanism of WalRK-mediated VISA development, via mutation to auxiliary proteins YycHI. Along with reduced vancomycin susceptibility, deletion of *yycH* or *yycI* resulted in reduced autolysis and downregulation of genes under the transcriptional control of WalRK, including *atlA*, *sle1* and CHAP domain amidase/peptidoglycan hydrolase genes SaurJH1_0704 and SaurJH1_2370 (Fig. 3B). Taken together these data suggest that in contrast to *Bacillus* sp., YycHI serves as an activator of WalRK in *S. aureus* and that the VISA phenotype associated with *yycHI* mutation correlates to reduced activity of the system, as opposed to enhanced activity, which was previously proposed^6^,^9^,^27^.

The regulatory impact of YycHI in *B. subtilis* was found to be as a result of direct interaction with the kinase, YycG^18^,^19^. A report investigating YycH and YycI from *S. aureus* failed to provide evidence of an interaction with WalK using a detergent-micelle-model^28^. Here, we have used a bacterial two-hybrid system to show that YycH or YycI in isolation was not sufficient to produce an interaction with WalK but when all three proteins were present, a ternary protein complex formed (Fig. 5B). This correlates with what has been described for *B. subtilis* using immunoprecipitation assays, whereby YycH only co-precipitated with YycG when YycI was present and vice versa^29^. YycH and YycI appear indispensible for the regulation of WalK, which explains why minimal transcriptional or phenotypic differences were observed between the *yycH*, *yycI* and *yycHI* deletion strains in this study. In prokaryotes, a number of TCRSs are regulated by interaction with auxiliary proteins. For example, the PII protein from *E. coli* acts as a repressor of NtrBC by blocking the autokinase domain and enhancing the phosphatase activity of the sensor kinase NtrB^30^. In addition, SaePQ serves as a repressor of the staphylococcal virulence regulator SaeSR by activating the phosphatase activity of the sensor kinase SaeS^31^. Whilst we have shown a physical interaction between YycHI and WalK, further experiments are required to understand exactly how YycHI serves to regulate WalRK in *S. aureus*.

The TMDs of YycH and YycI appeared to be important for protein-protein interactions (Fig. 5A). The *yycH* mutation associated with the clinical VISA strain, A8094 resulted in TMD truncation, which impacted on the interaction with YycI, as well as complex formation with WalK (Figs 1 and 5). Similarly, the *yycI* mutation associated with the clinical VISA strain, A6226 resulted in a frameshift within the TMD that led to similar effects upon interaction with YycH and WalK (Figs 1 and 5). Thus, based on current evidence, *yycHI* VISA mutations impact on WalRK activation as a direct result of reduced interaction with the sensor kinase WalK.

When compared to infections caused by VSSA, VISA are more likely to be associated with prolonged bacteremia and less likely to cause acute clinical instability such as septic shock^32^,^33^. In the current study, whilst we did not observe altered expression of major staphylococcal toxins, we did observe differential transcription of genes that impact on interaction with the host (Fig. 3C). Surface protein A (coded for by *spa*) is a trigger for platelet aggregation, which stimulates the production of platelet microbiocidal proteins^34^. Deceased expression of *spa*, as seen for our *yycH/I* deletion strains, likely results in reduced stimulation of platelet microbiocidal proteins that are important for host-defense against *S. aureus* bloodstream infection. As a corollary, increased expression of the capsule operon contributes to resistance to opsinophagocytosis by human polymorphonuclear leukocytes and enhanced bacterial persistence in a murine model of *S. aureus* infection^35^.

In keeping with the notion of YycHI as an activator of WalRK, the expression of Ig-binding B domain containing protein (*sbi*), complement inhibitors (*scn*, SaurJH1_1240) and fibrinogen binding proteins (*efb,* SauJH1_1235) were downregulated in this study, each of which have been shown to be positively regulated upon WalR overexpression^15^. Constitutive activation of WalR has also been shown to promote bacterial clearance and increase neutrophil recruitment *in vivo*^15^. Taken together, it is plausible that mutations to *yycHI* and reduced WalRK activation may contribute to a ‘stealth’ strategy coined by Gardete *et al*. whereby VISA alter the expression of virulence determinants to facilitate immune evasion and persistence within the context of infection^27^; a hypothesis that requires further exploration.

In summary, our data highlight a novel role for YycH and YycI to the development of reduced vancomycin susceptibility in *S. aureus*. YycHI mutations associated with VISA directly impacted upon interaction with the sensor kinase WalK and *yycH*/*yycI* deletion resulted in reduced expression of genes under its control. This indicates a positive role for YycHI proteins in regulating the WalRK TCRS in *S. aureus*.

## Methods

### Bacterial strains, plasmids and culture conditions

*S. aureus* strains were grown in Heart Infusion (HI) broth or agar and are listed in Table 1. *E. coli* strains were grown in LB broth or agar and are listed in Table S4. For the selection of strains carrying plasmids (listed in Table S4), media was supplemented with ampicillin (100 μg/mL), carbenicillin (100 μg/mL), kanamycin (50 μg/mL) or chloramphenicol (25 μg/mL).

### Genetic manipulation

The *E. coli/S. aureus* shuttle vector pKOR1 was used to generate in-frame deletions of *yycH* (A8090Δ*yycH*), *yycI* (A8090Δ*yycI*) and *yycHI* (A8090Δ*yycHI*) as described previously^36^. Approximately one kilobase up- and down-stream of each gene was amplified using primers listed in Table S4. Products were cloned into pKOR1 using BP Clonase (Invitrogen), then used to transform *E. coli* DH5α (NEB). Each plasmid was passaged through *S. aureus* strain RN4220 before being electroporated into A8090^37^. Integration of the vector was stimulated by growing cells in the presence of chloramphenicol at 42 °C followed by vector excision at 30 °C (no antibiotic). Counter-selection was achieved using 1 μg/mL anhydrotetracycline (ATc).

Gene reconstitution *in situ* was performed for A8090Δ*yycH* and A8090Δ*yycI* using the *E. coli*/*S. aureus* shuttle vector pIMAY^38^. Silent PvuI restriction sites were engineered into each reconstituted gene using splicing with overlap extension PCR (SOE-PCR)^38^,^39^. The reconstituted genes were introduced into pIMAY, generating pIMAY::*yycH* and pIMAY::*yycI* (Table S4) and the vectors were electroporated into A8090Δ*yycH* and A8090Δ*yycI,* respectively. Integration, plasmid excision and counter-selection were performed as described^38^ generating A8090::*yycH* and A8090::*yycI* (Table 1). To distinguish each strain from A8090, a 3.8 kb fragment encompassing *yycHI* was amplified from A8090, A8090::*yycH* and A8090::*yycI* and the products were digested with PvuI. A8090 produced one fragment whereas A8090::*yycH* and A8090::*yycI* each produced two (data not shown).

### Antibiotic susceptibility testing and autolysis assays

Minimum inhibitory concentrations (MICs) were determined using Etest (bioMérieux) as per manufacturer’s specifications. To assess for antibiotic heteroresistance, population analysis profiling was performed using the method of Wootton *et al*.^9^,^40^.

Autolytic activity was assessed using Triton X-100 induction^41^. *S. aureus* cells were grown to an optical density at 600 nm (OD_600_) of 0.8, chilled then pelleted by centrifugation. Cells were washed once with PBS then resuspended in PBS (pH 7.4) supplemented with 0.05% Triton X-100 to an OD_600_ of 1.0. Autolysis was defined as the decline in OD_600_ over time.

### RNA extraction, sequencing and digital PCR

For RNA sequencing, total RNA was extracted from early stationary phase (OD_600_ 7.0, Fig. S2B), as the impact of YycHI upon WalR-dependent gene expression in *Bacillus* sp. was most pronounced at this growth stage^19^. RNA was stabilized *in situ* using RNA*later* (QIAGEN) and samples were frozen at −80 °C. *S. aureus* cells were thawed at ambient temperature then disrupted using a Precellys 24 tissue homogenizer (Bertin Technologies). RNA was purified using an RNeasy mini kit (QIAGEN), with on-column DNAse I (QIAGEN) digestion. RNA from two independent extractions was forwarded to the Beijing Genomics Institute for sequencing using Illumina HiSeq 2000. Reads were mapped to the A8090 genome (GenBank accession NC_009632) and gene expression was quantified using the Reads Per Kilobase Per Million (RPKM) method^42^. Differential gene expression between strains was determined using NOISeq^43^. A fold change ratio of >2.0 with a q-value ≥0.8 was considered significant as described elsewhere^43^. To validate the differential gene expression data generated by RNA sequencing, digital PCR was performed for *sle1*, SaurJH1_2073, *capL*, *spa* and *efb* (primers listed in Table S5). cDNA (500 ng) was synthesized using Superscript III reverse transcriptase (Invitrogen). Digital PCR was performed as described by the manufacturer (Bio-Rad) for three biological replicates and gene expression was normalized against *gyrB* expression.

### Bacterial two-hybrid (BTH) analysis

Bacterial two-hybrid vectors were constructed and assays performed as described previously (Table S4)^44^. Coding sequences of proteins of interest were fused to either T25 (pKT25) or T18 (pUT18C) of adenylate cyclase. Each construct was verified by DNA sequencing. To determine if protein fragments interacted, combinations of pKT25 and pUT18C based plasmids were co-transformed into *E. coli* strain BTH101, which is adenylate cyclase deficient. Interaction was observed via functional complementation of adenylate cyclase and expression of the *lac* reporter^44^. The relative strength of interaction was quantified by measuring β-galactosidase activity using o-nitrophenol-β-galactoside (ONPG) as a substrate, as described elsewhere^45^. β-galactosidase assays were performed in triplicate for 4 biological replicates. Significance was determined by Mann-Whitney *U* test with a significance level of *P* < 0.05.

### Published during review process

While this paper was being reviewed, a manuscript was published by Poupel O *et al*. (Poupel *et al*., 2016 doi: 10.1371/journal.pone.0151449) that supported the key findings from this study. Deletion of *yycHI* from the *S. aureus* HG001 genetic background (vancomycin MIC 3 µg/ml) resulted in a decrease in vancomycin susceptibility (MIC 4 µg/ml) which was restored upon complementation. The authors concluded that the YycHI complex does not act as a repressor of WalRK in *S. aureus* by analyzing autolysis and the expression of *atlA, sle* and *saouhsc00773* in *yycH* and *yycI* deletion mutants during the exponential phase of growth (OD 600 nm 1.0). By assessing global gene expression at a later point in the growth cycle (OD 600 nm 7.0) we have now shown that in contrast to *Bacillus*, YycHI induces the expression of genes under the transcriptional control of WalRK in *S. aureus*.

## Additional Information

**How to cite this article**: Cameron, D. R. *et al*. Vancomycin susceptibility in methicillin-resistant *Staphylococcus aureus* is mediated by YycHI activation of the WalRK essential two-component regulatory system. *Sci. Rep.* **6**, 30823; doi: 10.1038/srep30823 (2016).

## Supplementary Material

Supplementary Information

## Figures and Tables

**Table 1:** Methicillin-resistant *S. aureus* strains used in this study.

Strain	Description	WalRK/YycHI mutation	MIC (μg/mL)		References
			VAN^a^	DAP	
*Clinical strains*					
A6224	VSSA	—	2.0	0.5	9
A6226	VISA derived from A6224	YycI missense from 27, premature stop at 56	3.0	2.0	9
A8090^b^	VSSA	—	1.0–1.5	0.5	6
A8094^c^	VISA derived from A8090	YycH W27stop	8.0	2.0	6
JKD6009	VSSA	—	1.5	0.19	16
JKD6008	VISA derived from JKD6009	WalK G223D	3.0	0.5	16
*Laboratory strains*					
A8117	VSSA	—	1.0	—	9
A8118	VISA derived from A8117	WalK R263C/S273N	4.0	—	9
A8392	VISA derived from A8117	WalK Q371Δ	8.0	—	9
A8090Δ*yycH*	A8090 with deletion of *yycH*	Δ*yycH*	2.0–3.0	0.5	This study
A8090Δ*yycI*	A8090 with deletion of *yycI*	Δ*yycI*	2.0–3.0	0.5	This study
A8090Δ*yycHI*	A8090 with deletion of *yycHI*	Δ*yycHI*	2.0–3.0	0.75	This study
A8090::*yycH*	A8090Δ*yycH* reconstituted with *yycH* carrying a silent PvuI restriction site	*yycH* silent PvuI site	1.0	—	This study
A8090::*yycI*	A8090Δ*yycI* with *yycI* carrying a silent PvuI restriction site	*yycI* silent PvuI site	1.0–1.5	—	This study

MIC, minimum inhibitory concentration; VAN, vancomycin; VISA, vancomycin-intermediate *S. aureus;* VSSA, vancomycin-susceptible *S. aureus*.

^a^MICs for vancomycin were determined 4 times independently for A8090 and its mutant derivatives (A8090Δ*yycH,* A8090Δ*yycI,* A8090Δ*yycHI,* A8090::*yycH* and A8090::*yycI*).

^b^Previously referred to as JH1^6^.

^c^Previously referred to as JH9^6^.
