# Supplementary material for: Vancomycin susceptibility in methicillin-resistant Staphylococcus aureus is mediated by YycHI activation of the WalRK essential two-component regulatory system
Source: Sci Rep. 2016 Sep 7;6:30823. doi: 10.1038/srep30823 (PMC5013275; doi:10.1038/srep30823)
Supplement: Supplementary Information [file srep30823-s1.doc]

**Vancomycin susceptibility in methicillin-resistant *Staphylococcus aureus* is mediated by YycHI activation of the WalRK essential two-component regulatory system**

**David R. Cameron^1^, Jhih-Hang Jiang^1^, Xenia Kostoulias^1^, Daniel J. Foxwell^1^, Anton Y. Peleg^1, 2*^**

^1^Department of Microbiology, Monash University, Melbourne, Australia and ^2^Department of Infectious Diseases, Alfred Hospital and Monash University, Melbourne, Australia.

**Supplementary Material**

**Table S1. Genes with altered expression in Δ*yycH* when compared to Δy*ycI*.**

| **Locus^a^** | **Gene** | **Product** | **Fold change** |
| --- | --- | --- | --- |
| *Down-regulated* | | | |
| SaurJH1_0217 | *pflA* | Pyruvate formate-lyase activating enzyme | 2.93 |
| SaurJH1_2470 | *nasF* | Uroporphyrin-III C-methyltransferase | 2.84 |
| SaurJH1_2471 | *nasE* | Nitrate reductase (NAD[P]H) small subunit | 2.90 |
| *Up-regulated* | | | |
| SaurJH1_2580 | *gntP* | Gluconate transporter | 12.66 |
| SaurJH1_2614 | *ptsG* | PTS system, glucose-specific IIBC subunit | 4.34 |
| SaurJH1_2689 | *gbsA* | Betaine aldehyde dehydrogenase | 2.63 |

^a^SaurJH1_ locus tag derived from the A8090/JH1 genome annotation.

**Table S2. Downregulated genes in Δ*yycH* and Δ*yycI*** when compared to A8090.

| **Locus^a^** | **Gene** | **Product** | **Fold change** | |
| --- | --- | --- | --- | --- |
|  |  |  | **Δ*yycH*** | **Δ*yycI*** |
| walKR *operon* | | | | |
| SaurJH1_0020 | *yycH* | Putative WalKR regulatory protein | 5316.6 | 1.18^b^ |
| SaurJH1_0021 | *yycI* | Putative WalKR regulatory protein | 1.08^b^ | 1016.4 |
| *Autolysis* | | | | |
| SaurJH1_0499 | *sle1* | CHAP domain-containing protein | 5.15 | 3.04 |
| SaurJH1_1135 | *atlA* | Bifunctional autolysin | 2.02^b^ | 2.34 |
| SaurJH1_0704 |  | CHAP domain-containing protein | 3.12 | 2.83 |
| SaurJH1_2370 |  | CHAP domain-containing protein | 6.72 | 9.96 |
| *Host-pathogen interaction* | | | | |
| SaurJH1_0102 | *spa* | Protein A | 3.67 | 4.24 |
| SaurJH1_2492 | *sbi* | Ig-binding B domain-containing protein | 2.50 | 2.06 |
| SaurJH1_1239 | *efb* | Fibrinogen binding protein | 3.37 | 2.57 |
| SaurJH1_1235 |  | Fibrinogen binding protein, putative | 2.10^b^ | 2.66 |
| SaurJH1_1240 |  | Staphylococcal complement inhibitor | 1.92^b^ | 2.67 |
| SaurJH1_2034 | *scn* | Staphylococcal complement inhibitor | 1.93^b^ | 2.04 |
| SaurJH1_2106 | *sdrH* | Serine-aspartate repeat protein H | 3.45 | 2.27 |
| *Other function* | | | | |
| SaurJH1_2403 | *lyrA* | Lysostaphin resistance protein A | 2.01^b^ | 2.08 |
| SaurJH1_1657 | *glyS* | Glycyl-tRNA synthetase | 1.35 | 2.05 |
| SaurJH1_0978 | *glpQ* | Glycerophosphodiester phosphodiesterase | 2.89 | 2.01 |
| SaurJH1_2589 |  | ABC transporter | 2.82^b^ | 2.77 |
| SaurJH1_2590 |  | Membrane spanning protein | 2.00^b^ | 2.42 |
| SaurJH1_1188 |  | Hypothetical protein | 1.99^b^ | 2.24 |

^a^SaurJH1_ locus tag derived from the A8090/JH1 genome annotation.

^b^Not significant.

**Table S3. Upregulated genes in Δ*yycH* and Δ*yycI*** when compared to A8090.

| **Locus^a^** | **Gene** | **Product** | **Fold change** | |
| --- | --- | --- | --- | --- |
|  |  |  | **Δ*yycH*** | **Δ*yycI*** |
| *Capsule biosynthesis* | | | | |
| SaurJH1_0140 | *capA* | MPA1 family polysaccharide export protein | 3.46 | 2.67 |
| SaurJH1_0141 | *capB* | Exopolysaccharide tyrosine-protein kinase | 3.02 | 2.6 |
| SaurJH1_0142 | *capC* | Protein-tyrosine-phosphatase | 4.06 | 2.86 |
| SaurJH1_0143 | *capD* | Polysaccharide biosynthesis protein CapD | 4.25 | 2.88 |
| SaurJH1_0144 | *capE* | Polysaccharide biosynthesis protein CapE | 4.73 | 2.44 |
| SaurJH1_0145 | *capF* | NAD-dependent epimerase/dehydratase | 4.41 | 1.77^b^ |
| SaurJH1_0146 | *capG* | UDP-N-acetylglucosamine 2-epimerase | 3.97 | 1.82^b^ |
| SaurJH1_0151 | *capL* | Group 1 glycosyl transferase | 4.98 | 2.97 |
| SaurJH1_0152 | *capM* | Sugar transferase | 4.5 | 2.75 |
| SaurJH1_0153 | *capN* | NAD-dependent epimerase/dehydratase | 4.79 | 2.78 |
| SaurJH1_0154 | *capO* | UDP-glucose/GDP-mannose dehydrogenase | 3.68 | 2.61 |
| SaurJH1_0155 | *capP* | UDP-N-acetylglucosamine 2-epimerase | 4.08 | 2.49 |
| *Amino acid biosynthesis* | | | | |
| SaurJH1_2131 | *leuA* | 2-isopropylmalate synthase | 2.19^b^ | 2.28 |
| SaurJH1_2132 | *leuB* | 3-isopropylmalate dehydrogenase | 1.5^b^ | 2.41 |
| SaurJH1_2133 | *leuC* | Isopropylmalate isomerase large subunit | 2.13^b^ | 2.28 |
| SaurJH1_2134 | *leuD* | Isopropylmalate isomerase small subunit | 2.07^b^ | 2.45 |
| *Choline metabolism* | | | | |
| SaurJH1_2688 | *betA* | Choline dehydrogenase | 5.61 | 3.05 |
| SaurJH1_2689 | *gbsA* | Betaine aldehyde dehydrogenase | 7.19 | 2.72 |
| *Other function* | | | | |
| SaurJH1_0812 | *gapR* | Glyocolytic operon regulator | 2.25^b^ | 2.69 |
| SaurJH1_0224 |  | AMP-dependent synthetase and ligase | 1.82^b^ | 2.11 |
| SaurJH1_0308 |  | N-acetylmannosamine-6-phosphate 2-epimerase | 1.37^b^ | 2.23 |
| SaurJH1_2580 | *gntP* | Gluconate permease | 16.50 | 1.30^b^ |
| SaurJH1_2614 | *ptsG* | PTS system, glucose-specific IIBC subunit | 4.99 | 1.16^b^ |
| SaurJH1_1130 | *sspA* | Serine protease, glutamyl endopeptidase | 2.95 | 1.86^b^ |
| SaurJH1_2274 |  | MAP domain-containing protein | 3.08 | 1.48^b^ |

^a^SaurJH1_ locus tag derived from the A8090/JH1 genome annotation.

^b^Not significant.

**Table S4. *E. coli* strains and plasmids** used in this study

| **Strain** | **Description** | **Reference** |
| --- | --- | --- |
| *E. coli* | | |
| DH5α | General cloning strain | NEB |
| DC10B | Dam methylation deficient derivative of DH10B | 1 |
| BTH101 | *E. coli* host for BTH analysis | 2 |
| *Plasmids* | | |
| pKOR1 | Amp^R^; Cm^R^; *S. aureus*/*E. coli* shuttle vector | 3 |
| pKOR1Δ*yycH* | *yycH* deletion construct | This study |
| pKOR1Δ*yycI* | *yycI* deletion construct | This study |
| pKOR1Δ*yycHI* | *yycHI* deletion construct | This study |
| pIMAY | Cm^R^; *S. aureus*/*E. coli* shuttle vector | 1 |
| pIMAY::*yycH* | *yycH* with silent PvuI site gene reconstitution vector | This study |
| pIMAY::*yycI* | *yycI* with silent PvuI site gene reconstitution vector | This study |
| pKT25 | Kan^R^; T25 subunit of *cyaA* | 2 |
| pUT18C | Cb^R^; T18 subunit of *cyaA* | 2 |
| pUT*yycI* | T18 fused to *yycI* | This study |
| pKT*yycI* | T25 fused to *yycI* | This study |
| pKT*yycI*_1-53_ | T25 fused to *yycI*_1-53_ | This study |
| pKT*yycI*_36-262_ | T25 fused to *yycI*_36-262_ | This study |
| pKT*yycI*_A6226_ | T25 fused to *yycHI* from A6226 (VISA) | This study |
| pKT*yycH* | T25 fused to *yycH* | This study |
| pUT*yycH* | T18 fused to *yycH* | This study |
| pUT*yycH*_1-36_ | T18 fused to *yycH*_1-36_ | This study |
| pUT*yycH*_40-444_ | T18 fused to *yycH*_40-444_ | This study |
| pUT*yycH*_A8094_ | T18 fused to *yycHI* amplified from A8094 (VISA) | This study |
| pKT*walK* | T25 fused to *walK* | This study |
| pUT*yycHI* | T18 fused to *yycHI* | This study |
| pUT*yycHI*_A8094_ | T18 fused to *yycHI* from A8094 (VISA) | This study |
| pUT*yycHI*_A6226_ | T18 fused to *yycHI* from A6226 (VISA) | This study |

Amp^R^, ampicillin resistance; Cb^R^, carbenicillin resistance; Cm^R^, chloramphenicol resistance; MRSA, methicillin-resistant *S. aureus*

**Table S5**: Oligonucleotide primers used in this study.

| **Function** | **AP #** | **Sequence (5'  3')** |
| --- | --- | --- |
| *yycI* deletion | 158 | *att*B1_CAGAAGGTACGATTGCGACG |
|  | 159 | CCCCCGCGGGTGTCTTTGTCAGTTTCCAG |
|  | 160 | CCCCCGCGGCTTACTATGTCGAAGCGAC |
|  | 161 | *att*B2_GCTGCCACGTATCATACC |
| *yycH* deletion | 162 | *att*B1_GAAGAAGGTCTAATCGCACG |
|  | 163 | CCCCCGCGGCATCAAGACGAGTAGCGC |
|  | 164 | CCCCCGCGGCCGCGTTGGTATGTAGAATATG |
|  | 165 | *att*B2_CTTTAGTTCATCGACCGG |
| *yycH* complement | 356 | CCCGAATTCCGTAGACTGGACTCAGTTATCACC |
|  | 357 | CTTTGAACTACGAACGATCGTTGAATCGTC |
|  | 358 | CTATTTGACGATTCAACGATCGTTCGTAG |
|  | 359 | CCCGGTACCCTCATGCGTATCAAGCGGCTC |
| *yycI* complement | 360 | CCCGAATTCGTAAAGACCGCCACGATTACG |
|  | 361 | CTTTAGAACTAAAGTCTTTCGATCGCCCTG |
|  | 362 | GCAAATTACAGGGCGATCGAAAGACTTTAG |
|  | 363 | CCCGGTACCCAGCAGTGCCGAACTTTATAGGGTG |
| *yycH* B2H | 364 | CCCTCTAGAGTCATTGAAGACGGTGATTGGGATG |
|  | 365 | CCCGGATCCCCAGTTCATTTATTCAAGCCTCCC |
|  | 629 | CCCGGATCCCTAGACATTTGCAATATCAGGAG |
|  | 630 | CCCTCTAGATCCTGATATTGCAAATGTCGAC |
| *yycI* B2H | 366 | CCCTCTAGAGAACTGGAAACTGACAAAGACAC |
|  | 367 | CCCGGATCCCCGCTTCTCACATATGATGAGC |
|  | 790 | CCCGGATCCTTACGGTACTTTAATTTCTTCTTGCTG |
|  | 630 | CCCTCTAGATCCTGATATTGCAAATGTCGAC |
| w*alK* B2H | 368 | CCCGTCGACGAAGTGGCTAAAACAACTACAATCCC |
|  | 369 | CCCGGATCCCATCCCAATCACCGTCTTCAATGAC |
|  | 791 | CCCGGATCCTTACATTTCGACCGTCTGGTTACG |
|  | 792 | CCCGGATCCTTAGGTGATAACTGAGTCCAGTCTACG |
|  | 793 | CCCGGATCCTTACTCACGTTCAACTTGTTGTTGTTC |
|  | 794 | CCCGGATCCTTAGTCTTTCGCAGACATTTCATGTCG |
| *sle1* digital PCR | 505 | CTGCTAGCTCAAGTAACGCTG |
|  | 506 | GACAATGAGTCACCTGCTTG |
| SaurJH1_2370 digital PCR | 507 | CAACACGACTCAAACTACAACGAC |
|  | 508 | CCAAGTACATTGTCCTGCAGTG |
| *gyrB* digital PCR | 509 | CCGATTGCTCTAGTAAAAGTCCTG |
|  | 510 | CGTAATGGTAAAATCGCCTGC |
| *capL* digital PCR | 650 | ACGGCAATAGAAGACCATCG |
|  | 651 | CGACGCCTTCTTTAATCAGC |
| *spa* digital PCR | 652 | CAAACGGCACTACTGCTGAC |
|  | 653 | TTAGCATCTGCATGGTTTGC |
| *efb* digital PCR | 654 | TAAACCAGCAGCGAAAACTG |
|  | 655 | CTAAGTTGACTGCCTTTTGTGC |
| SaurJH1_2034 digital PCR | 657 | ACTTGCGGGAACTTTAGCAATC |
|  | 658 | TTCATTCGATGTTGGCAAGC |

B2H, bacterial-two hybrid

Note: attB1, GGGGACAAGTTTGTACAAAAAAGCAGGCT;

attB2, GGGGACCACTTTGTACAAGAAAGCTGGGT


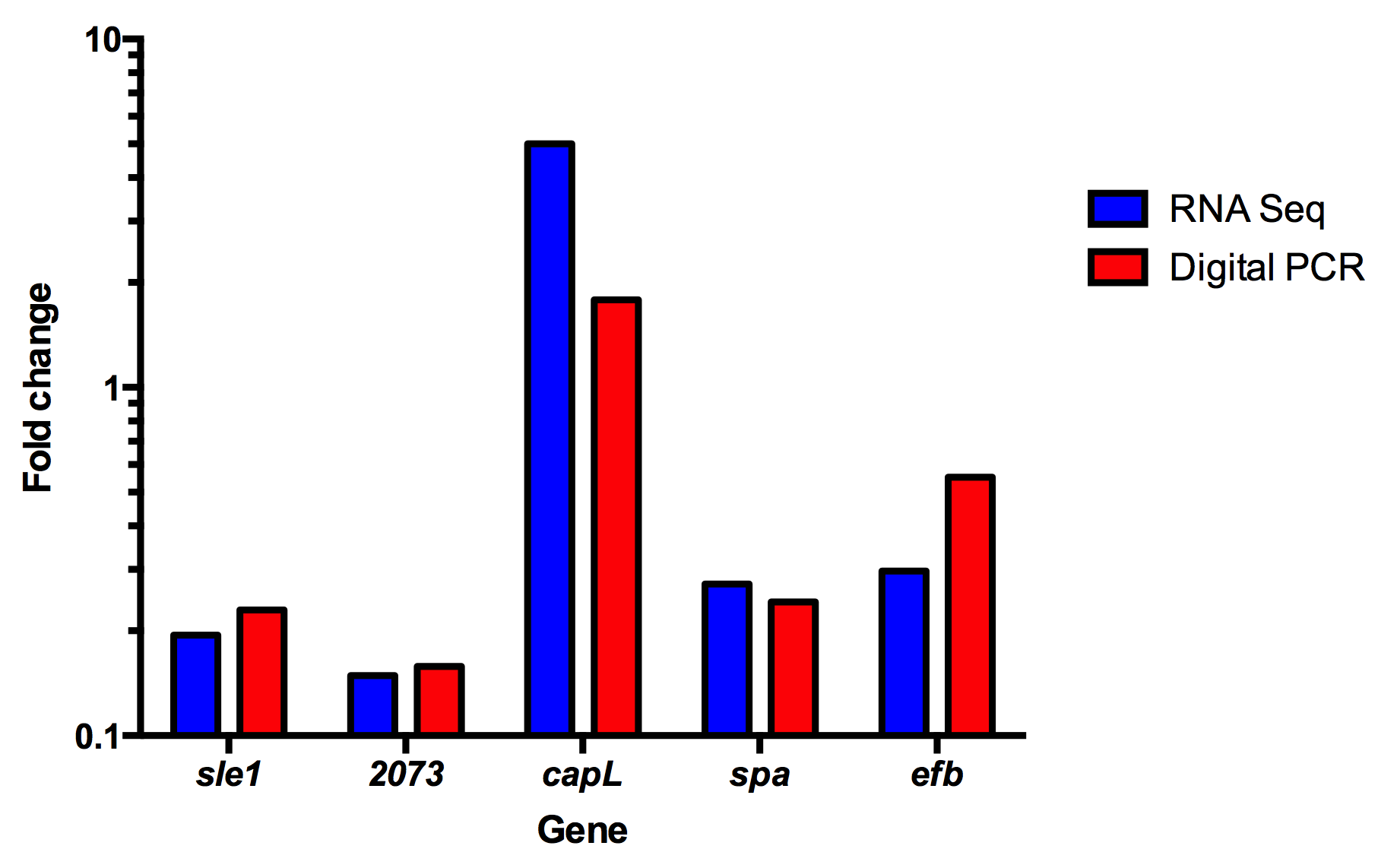


**Figure S1: Validation of RNA-Seq data using digital PCR.** The fold change ratios for five genes found to have altered expression between A8090 and *ΔyycH* using RNA-Seq were similar when determined using quantitative digital PCR.

**
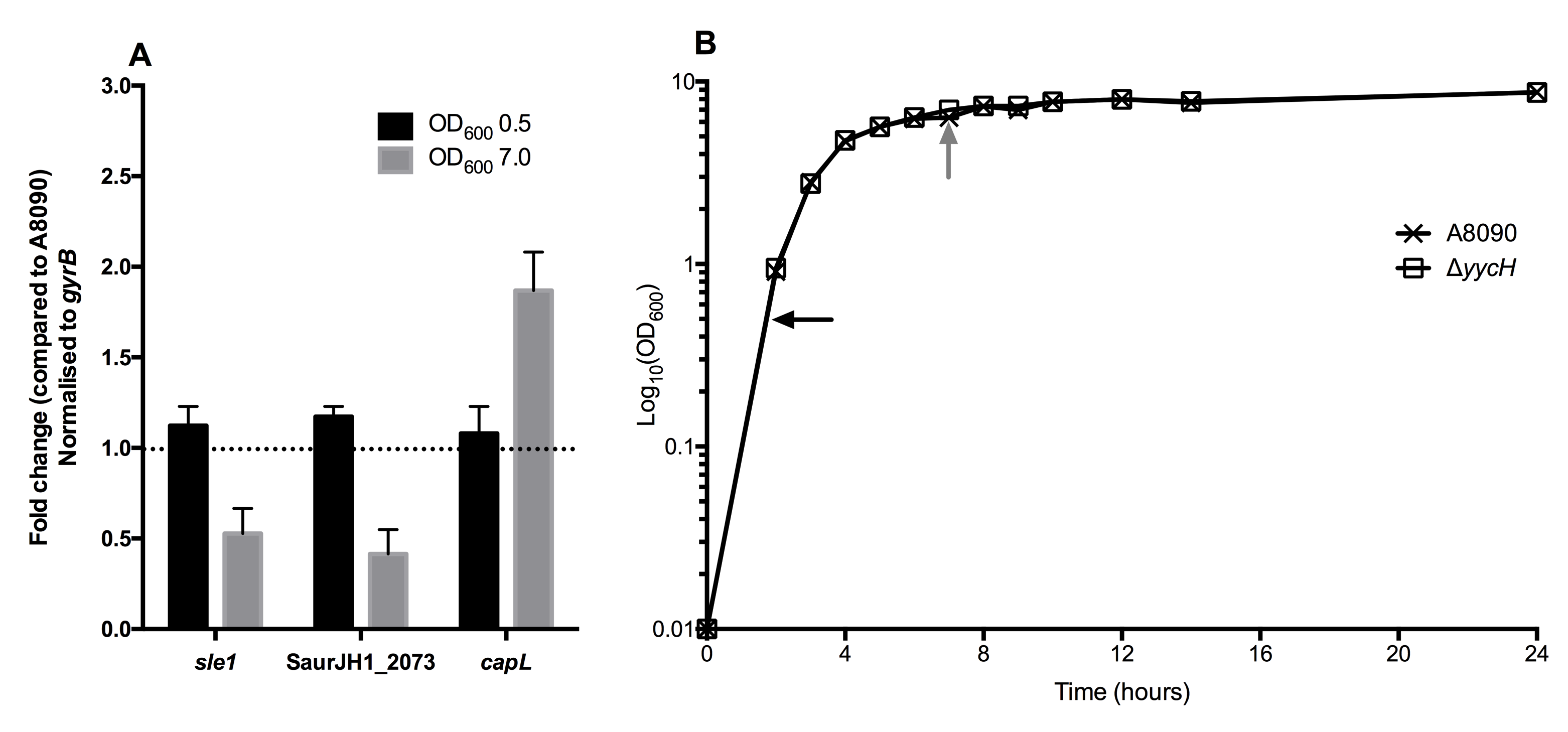
**

**Figure S2: Growth phase dependent transcriptional changes for Δ*yycH* when compared to A8090. (A)** Differential expression of WalR-dependent genes (*sle1* and SaurJH1_2073) and a representative gene from the capsule operon (*capL*) was more pronounced at stationary phase (OD_600_ 7.0) when compared to exponential phase (OD_600_ 0.5) as determined by quantitative digital PCR. **(B)** Growth curve comparison of A8090 and *ΔyycH* grown in HI media at 37°C. The black arrow represents the point at exponential phase where RNA was extracted for digital PCR (OD 0.5). The grey arrow represents the point at early-stationary phase where RNA-Seq analysis and digital PCR was performed (OD 7.0).

**References**

1 Monk, I. R., Shah, I. M., Xu, M., Tan, M. W. & Foster, T. J. Transforming the untransformable: application of direct transformation to manipulate genetically *Staphylococcus aureus* and *Staphylococcus epidermidis*. *mBio* **3**, doi:10.1128/mBio.00277-11 (2012).

2 Karimova, G., Pidoux, J., Ullmann, A. & Ladant, D. A bacterial two-hybrid system based on a reconstituted signal transduction pathway. *Proc. Natl. Acad. Sci. U. S. A.* **95**, 5752-5756 (1998).

3 Bae, T. & Schneewind, O. Allelic replacement in *Staphylococcus aureus* with inducible counter-selection. *Plasmid* **55**, 58-63 (2006).
